# Effectiveness of theta and gamma electroacupuncture for post-stroke patients on working memory and electrophysiology: study protocol for a double-center, randomized, patient- and assessor-blinded, sham-controlled, parallel, clinical trial

**DOI:** 10.1186/s13063-020-04807-z

**Published:** 2020-11-04

**Authors:** Jing-Jing Xu, Meng Ren, Jing-Jun Zhao, Jia-Jia Wu, Si-Cong Zhang, Yan-Biao Zhong, Shu-Tian Xu, Zhong-Yao Cao, Zhi-Qing Zhou, Yuan-Li Li, Chun-Lei Shan

**Affiliations:** 1 Yueyang Hospital of Integrated Traditional Chinese and Western Medicine, Shanghai University of Traditional Chinese Medicine, 110 Ganhe Road, Hongkou District, Shanghai, 200000 China; 2 Shanghai University of Traditional Chinese Medicine, 1200 Cailun Road, Pudong New District, Shanghai, 200000 China; 3 Anhui Wannan Rehabilitation Hospital, 3 Zheshang road, Jinghu District, Wuhu City, 241000 Anhui Province China

**Keywords:** Post-stroke, Working memory (WM), Electroacupuncture (EA), Randomized controlled trial

## Abstract

**Background:**

Practitioners of complementary and alternative medicine have suggested that electroacupuncture (EA) could improve post-stroke cognitive impairment, based on the clinical evidence. This study protocol is aimed at showing the effectiveness of theta and gamma EA for post-stroke patients on working memory (WM) and electrophysiology.

**Methods:**

After assessing their eligibility, 66 patients with stroke will be enrolled from two Chinese medicine hospitals and randomly divided into theta frequency EA group, gamma frequency EA group, and sham-EA group according to the ratio of 1:1:1. All patients will receive 20 sessions of EA procedures for 4 weeks. Patients in three groups will receive EA at two same acupoints in the head: Baihui (GV20) and Shenting (GV24). The frequency of the three groups of EA is set as follows: 6 Hz (theta-EA group), 40 Hz (gamma-EA group), and no current through the electrodes (sham EA). Patients and assessors will be blinded throughout the entire study. The primary outcome is the performance accuracy of 1-back task which is a frequently used measure of WM in cognitive neuroscience research contexts. Secondary outcome measures will include the response time of 1-back task, the Rivermead Behavioral Memory Test, Trail Making Test, Loewenstein Occupational Therapy Cognitive Assessment Scale, modified Barthel Index, and electroencephalogram (EEG) signals during 1-back tasks. A blinding index will be assessed. Data will be statistically analyzed by one-way ANOVA, at 5% of significance level.

**Discussion:**

We expect this double-center, randomized, patient- and assessor-blinded, sham-controlled, parallel, clinical trial to explore the effectiveness of theta and gamma EA therapy, compared with sham EA, for post-stroke WM.

**Trial registration:**

Chinese Clinical Trial Registry, ChiCTR2000031995. Registered on 17 April 2020.

## Background

Cognitive dysfunction is one of the common dysfunctions in patients after stroke. The decline in cognitive function has a serious impact on patients’ quality of life and is a major factor to prevent patients from returning to society [1, 2]. Working memory (WM), an important part of cognitive function, is considered to be the basis for the brain to successfully perform complex behaviors [3]. Studies have shown that the incidence of WM disorders among post-stroke patients is as high as 87.6% [4].

As the main responsibility for information retention and processing in the brain for a short time [5], WM decline will cause a decline in overall cognitive function [3, 6]. For example, patients with WM impairment may have difficulty performing daily activities such as memorization, reading, writing, planning, coherent performances, and communication [7, 8]. For post-stroke patients, WM disorder can also reduce the efficiency of individual rehabilitation training, such as learning and maintaining new movements [9]. In addition, emotional disequilibrium and self-regulation (such as depression) may also occur due to WM deficits [8, 10]. Despite they have a high incidence in stroke patients, WM disorders are often overlooked because they are hidden [4]. Therefore, there are still few clinical studies on WM rehabilitation after stroke [11].

Acupuncture, as the main treatment method of traditional Chinese medicine, is commonly used in the clinical treatment of neurological diseases [12–14]. The method of executing electrical stimulation on the needle body after inserting the needle into the acupoints is called electroacupuncture (EA) [15]. A meta-analysis has shown that acupuncture (including EA) has a positive effect on post-stroke cognitive function [16]. Nevertheless, can EA improve WM impairment after stroke? What is the optimal frequency of EA to improve WM? It is true that we need more related clinical researches on those questions.

Current neuroscience research has found that neural oscillations play an important role in a range of cognitive function [17–21]. For example, electroencephalogram (EEG) recording shows that subjects’ oscillations in theta (3–8 Hz) and gamma (> 30 Hz) band have a special correlation with WM [22–25]. Among them, the oscillations in the theta band are responsible for organizing and arranging WM items in sequence [26]; the oscillations in the gamma band play a role in maintaining WM information [27]. In addition, many experiments have confirmed that transcranial alternating current stimulation (tACS) at theta and gamma frequencies can enhance WM in healthy subjects [18–20].

Compared with tACS, EA is a more mature and widely used clinical treatment therapy. A large number of studies have confirmed the safety and effectiveness of EA in the treatment of post-stroke patients. Collectively, this study will explore the effects of EA in the theta (6 Hz) and gamma (40 Hz) bands on WM of stroke patients and investigate changes of electrical activity in the cerebral cortex before and after intervention.

## Methods/design

### Primary objective and study hypothesis

The main purpose of this clinical trial is to observe the efficacy of EA at theta, gamma, and sham frequencies on WM. We adopt the classic evaluation paradigm of WM: using 1-back paradigm to evaluate the performance of all patients before and after intervention. Three groups of patients were compared between baseline and after intervention. It is hypothesized that EA intervention at theta and/or gamma frequencies may significantly improve patients’ WM.

### Secondary objectives

(1) Since the central executive system is an important part of WM, the study will observe changes in executive functions. (2) WM is closely related to the patient’s overall cognitive level and ability to daily life. The study will also observe patients’ behavioral memory, overall cognition level, and daily life ability. (3) By using EEG, we recorded the electrical signals of the cerebral cortex of patients during 1-back tasks. Through the analysis of the EEG data, the effects of theta-EA and gamma-EA on the electrophysiological properties of the brain will be investigated and compared with sham EA.

### Study design

This study is a two-center, randomized, sham-controlled, blinded patient and evaluator, parallel clinical trial. Our study will show if it is feasible to arrange patients randomly in EA trials of WM after stroke. The results of the study will be used to sample size calculation to provide a basis for subsequent research.

Sixty-six post-stroke patients will be recruited to participate in the trial. Before the experiment begins, the doctor will provide specific information about the trial to patients both orally and in writing. All patients will voluntarily sign an informed consent provided by the attending doctor. The informed consent was approved by the ethics committee before the formal experiment begins. All patients will be screened according to eligibility criteria. Sixty-six post-stroke patients will be randomly divided into theta-EA group, gamma-EA group, and sham-EA group according to the ratio of 1:1:1. The EA treatment and evaluation did not perform simultaneously. The evaluation will be performed the day before the intervention. The second evaluation is at least 2 h after the last intervention. All patients will receive an intervention that lasts for 4 weeks (30 min a day, 5 days a week, Monday to Friday).

**Fig. 1:**
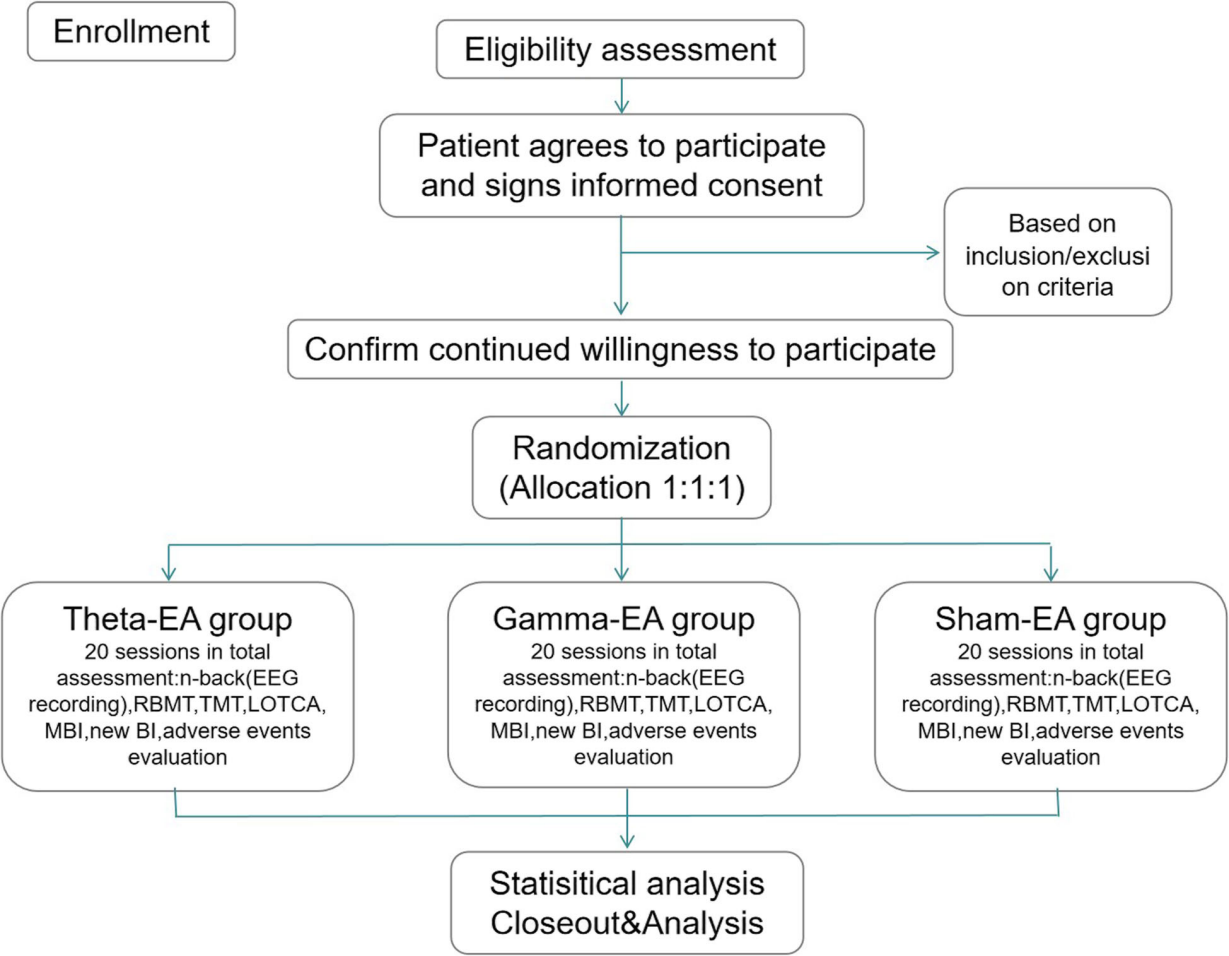
Flow diagram of the study design including the process of recruitment, allocation and intervention, and assessment. EA, electroacupuncture; RBMT, The Rivermead Behavioral Memory Test; TMT, Trail Making Test; LOTCA, Loewenstein Occupational Therapy Cognitive Assessment Scale; MBI, Modified Barthel Index; new BI, new blind index

**Fig. 2:**
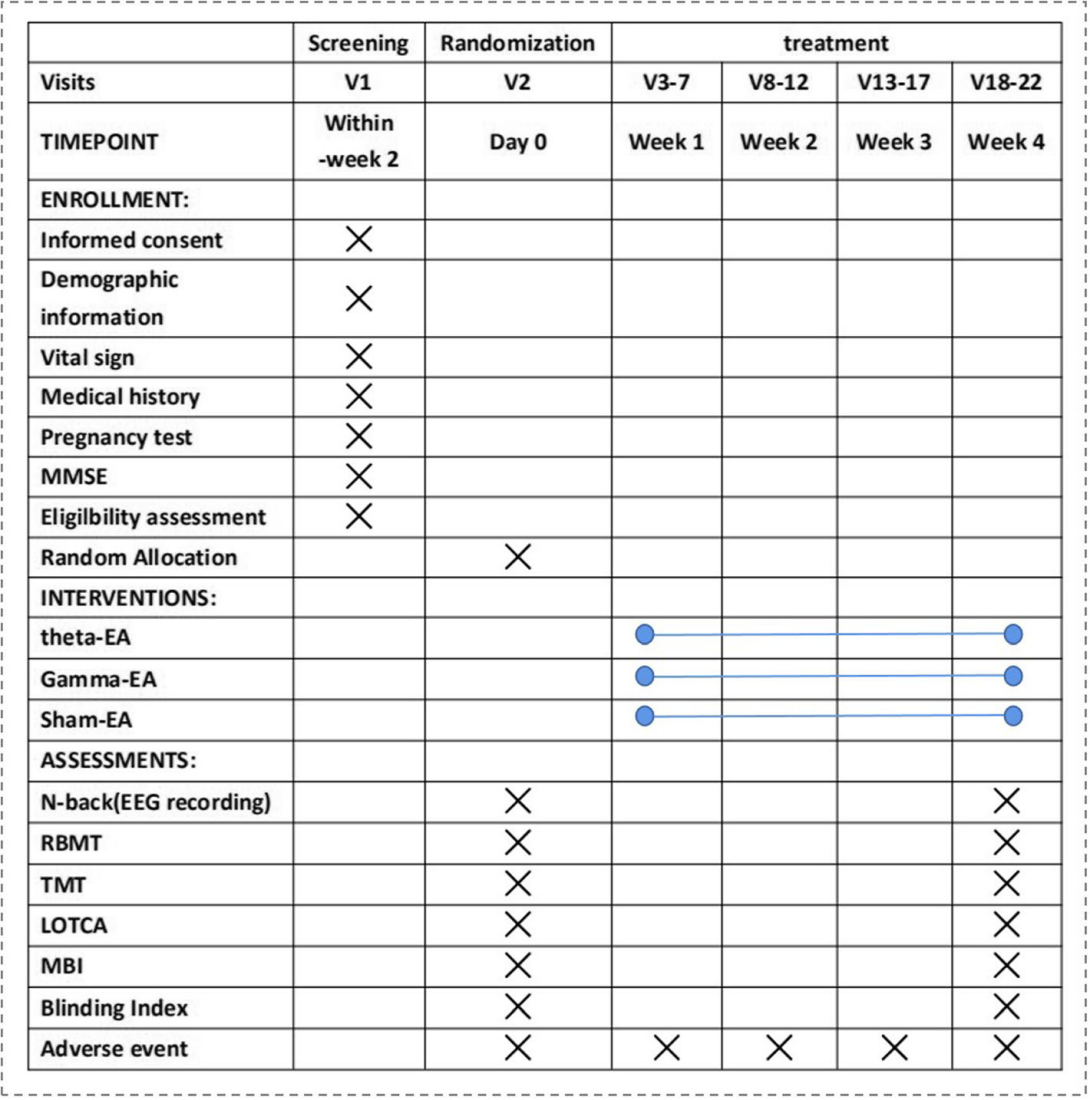
Standard Protocol Items: Reccommendations for Interventional Trials (SPIRIT) figure. EA, electroacupuncture; MMSE, Mini-Mental State Examination; RBMT, The Rivermead Behavioral Memory Test; TMT, Trail Making Test; LOTCA, Loewenstein Occupational Therapy Cognitive Assessment Scale; MBI, Modified Barthel Index; new, BI new blind index

Considering that the focus of this experiment is to explore the effects of EA at different frequencies on WM, patients in three groups will receive EA at two same acupoints in the head: Baihui (GV20) and Shenting (GV24). The frequency of the three groups of EA is selected as follows: 6 Hz (theta-EA), 40 Hz (gamma-EA), and no current through the electrodes (sham EA). The overall schematic diagram and schedule for the study are respectively shown in Figs. 1 and 2.

### Setting and recruitment

Eligible patients will be recruited by Yueyang Hospital of Integrated Traditional Chinese and Western Medicine affiliated to Shanghai University of Traditional Chinese Medicine and Anhui Wannan Rehabilitation Hospital. Both hospitals will post research advertisements online (website) and offline (poster) to recruit patients. To enhance recruitment quality, investigators will monitor medical records. The attending doctor will carefully inquire and record the patients’ past and present medical history.

### Ethical review and general statements

The study will be conducted in accordance with the research protocol and the current version of the Helsinki Declaration. All patients will be fully informed about the trial and sign a written informed consent form before participating. This trial was approved by the Ethics Committee of Yueyang Integrated Hospital of Traditional Chinese and Western Medicine, Shanghai University of Traditional Chinese Medicine (approval number: 2019-121), and registered in China Clinical Trial Registration Center (ChiCTR. gov) in March 2020 (registration number: ChiCTR2000031995).

### Eligibility criteria

#### Inclusion criteria

Eligible patients must meet all of the following criteria before being randomized: (a) male or female between the ages of 18 and 80; (b) diagnosis of cerebral hemorrhage or cerebral infarction by using computed tomography (CT) or magnetic resonance imaging (MRI); (c) got at least 24 points in MMSE test to ensure that the patient has not caught dementia; (d) had a stroke more than 1 month before; (e) GDS self-rating depression scale ≤ 6 points; (f) can tolerate an evaluation that lasts 2 h; (g) is consciousness and has a stable physical condition; and (h) giving verbal and written consent that signed and confirmed by the patient.

#### Exclusion criteria

Patients will be excluded if they meet one or more following criteria: (a) has severe visual and hearing problems; (b) has severe impairment in language understanding which shown by tests for aphasia screening scales; (c) has local skin infections, ulcers, and scars or is allergic to acupuncture; (d) has severe bleeding and coagulopathy; (e) body or head metal implants (except oral cavity): implantation of pacemakers, defibrillators, cochlear implants, and drug pumps; (f) skull fractures and/or severe head injury; (g) get seizures; (h) has mental disorders; (i) severe physical conditions (such as heart disease); and (j) other diseases or medical conditions that affect cognitive function.

### Randomization and allocation

For randomization, to reduce the chance of bias and confusion and increase the probability of meeting statistical analysis assumptions, a restricted randomization with a random block size between 3 and 6 will be used (the exact size is determined by an external agency) [28]. Two centers will both receive a computer-generated random list from third-party partners (MATLAB 2012b, Mathworks Inc., Natick, MA, USA) which will not participate in this research. Envelopes with sequential numbers are opaque and sealed and will be distributed to each test center by a third-party agency that is not part of the research team. Before the first treatment and after the treatment is completed, the therapist will be notified to perform a baseline and post-treatment evaluation of the patient.

### Blinding

To minimize deviations and disturbances caused by other factors, the acupuncturist will not give the patient any information about the treatment. In this study, the interventions that patients received in three groups will keep as consistent as possible except for different frequencies. Even needles’ position, angle, depth, and acupuncture technique that patients received will be kept completely consistent. Visually, the sham-EA group looks the same as the two observation groups. The electrodes will be connected to the needle body, and the EA instrument will also be turned on and modulated with the parameters. During the three groups of treatments, the EA instrument was placed on the back of the patient to ensure that the patient could not see the parameters of the stimulation. The specific stimulation scheme of sham EA will be introduced in the chapter of fake EA. The above scheme ensures that patients do not know what kind of intervention they are receiving. At the same time, assessors are not involved in the treatment to ensure the completion of the blind method. At the end of the study, patients and assessors will be asked to try to infer which group they think they belong to. Judgment results are calculated through new blindness index (BI) [29] to assess the success or failure of blinding. Unblinding should be determined on a case-by-case basis and considered only in a critical medical emergency. If unblinding happen, the subject will be terminated from the trial immediately, and the investigator will record the reason for the termination in the CRF. All data will be retrieved with the CRF after the trial, so as to analyze the reason, scope, and time of unblinding to evaluate the efficacy and safety of the study.

### Interventions

Patients will be divided equally into theta-EA, gamma-EA, or sham-EA. All groups will receive conventional neurorehabilitation treatment. The trial’s intervention program will be added to the existing treatment plan for 30 min per day for 4 weeks (Monday to Friday). To ensure adherence to intervention, we will strengthen the communication between researchers and patients such as researchers will communicate with patients to determine the time point for daily intervention to ensure that treatment is received at a fixed time. The monitor committee will control procedures for monitoring adherence that two trial centers are required to track and record specific rehabilitation programs and accurate information such as the frequency and dose of medications.

#### Conventional therapy

All patients will receive basic treatment, including daily rehabilitation and clinical medications. Conventional rehabilitation treatments include physical therapy and occupational therapy. During the intervention, the patient cannot receive other acupuncture treatments or take cognitive drugs. Two trial centers are required to track and record specific rehabilitation programs and accurate information such as the frequency and dose of medications.

#### Verum EA stimulation (theta-EA group, gamma-EA group)

Patients will be treated by an experienced acupuncturist with at least 2 years of clinical experience in acupuncture. The acupuncture points selected for treatment are [Baihui (GV20)] and [Shenting (GV24)]. Before treatment, the skin at the acupoint will be disinfected with 75% ethanol. And then the acupuncturist will use one hand to fix the patients’ head and use the other hand to insert the needle into the acupuncture point (Hwato, disposable, 0.35 × 40 mm, the distributor is Suzhou Hwato Medical Devices, Suzhou, China). The insertion angle is about 10–20° (between the needle and the scalp), and the insertion depth of the needle is about 0.3–0.5 cun (1 cun = 3.33 cm). After insertion, the acupuncturist will gently twist the needle for 1 min. After “Deqi”(a feeling of soreness, tingling, or bloating), the electrode will be connected to the needle body. Then the acupuncturist will set the stimulation parameters: the theta-EA group’s stimulation frequency is 6 Hz; the gamma-EA group’s stimulation frequency is 40hz [19]. The EA device (Hwato, SDZ-III, Suzhou, China) is placed behind the patient to ensure that the patient does not know the specific frequency parameters of the intervention. Patients should receive an intervention last for 4 weeks (30 min a day, 5 days a week, Monday to Friday) [12]. During the intervention, patients are asked to be quiet to minimize other interference factors.

#### Sham-EA group

The acupuncture points and operation procedures of the sham stimulation group were consistent with those of the other two groups. The only difference from the other two groups is that the electrode connected to the needle body is actually in a no-current state [30]. The details are as follows: (1) in the sham-EA group, a special wire which cannot be powered was used, although its appearance is not different from other ordinary wires; (2) visually, the sham-EA group looks the same as the two observation groups; (3) the EA device is placed behind the patient to ensure that the patient does not know the specific frequency parameters of the intervention; and (4) when the patient is undergoing intervention, the researcher will accompany the patient to ensure that the patient looks forward and stays still in a quiet state.

#### Dropout criteria

Patients will be discontinuing interventions if they meet one or more following criteria: (a) mistakes/acceptances (subjects who were mistakenly included); (b) subjects have poor compliance and have not been treated as prescribed, for example, subjects who cannot cooperate with researchers or subjects who are unable to remain quiet and stay still or subjects who cannot come to treatment on time; (c) incomplete medical record data that affects the evaluation of efficacy; (d) subjects withdraw on their own; (e) subjects have adverse events (including recurrence of stroke or cerebral hemorrhage, stuck needles, bent needles, hematoma, pain, and metal allergy); (f) the subject’s disease has worsened severely, or some comorbidities, complications, and special physiological changes have occurred (which is unknown adverse events that we may encounter during the study); and (g) the researcher believes that the subject is not suitable to continue participating in this study.

### Outcomes

#### Primary outcome

##### Accuracy (d`) scores of visualizing 1-back digital tasks

**Fig. 3:**
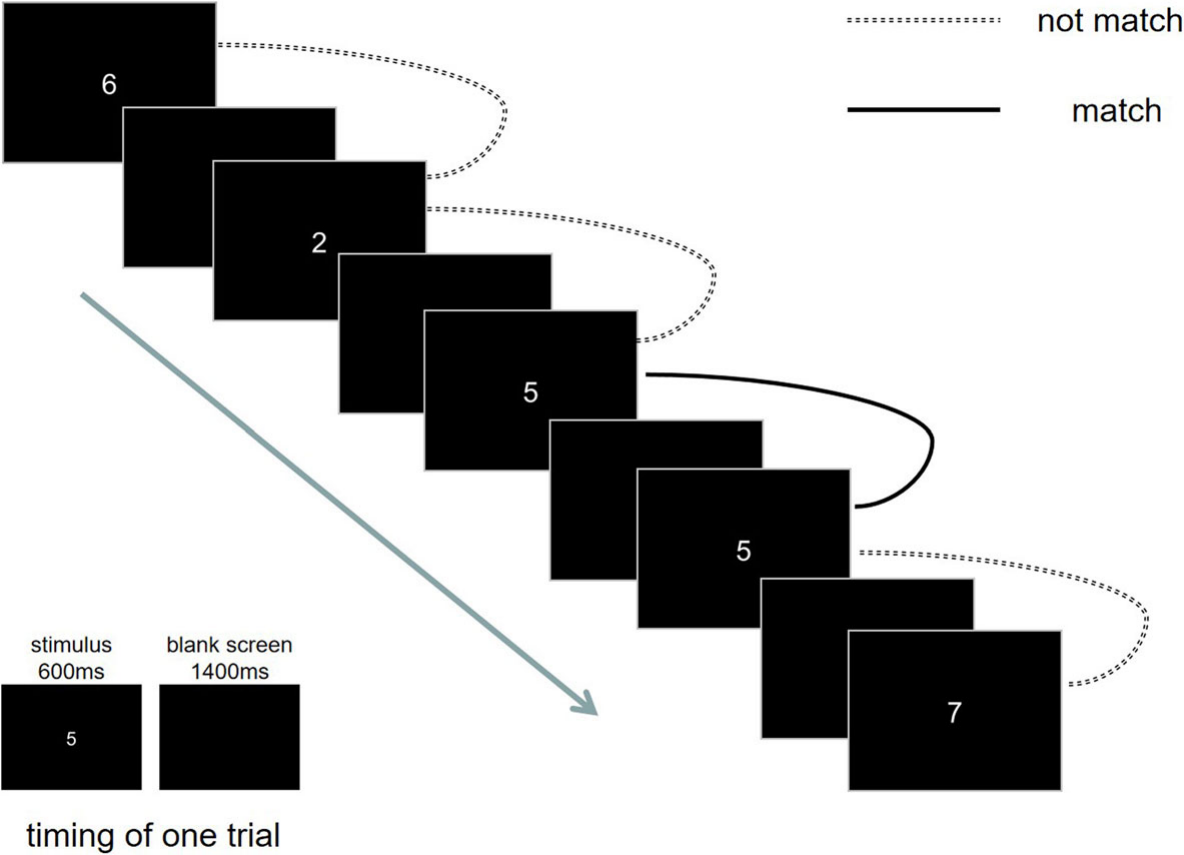
Illustration of 1-back task

The calculation of d` in 1-back will be described in detail in the statistics section. The patient will need to perform a visual 1-back digital task in 5 min. The patient will be asked to respond to stimuli (number shown on screen) as needed. In the 1-back, participants had to base their target/nontarget (i.e., match/not match) decision on the particular digital they saw within the sequence one trial before. The patient will be asked to press j on the keyboard as soon as possible if the numbers are the same while press f if not. This test includes 200 trials which include 40 target stimuli. Each stimulus will last for 600 ms, and then a blank screen of 1400 ms will be used as the stimulus interval [31]. The mission was edited and implemented by E-prime 3 and will be displayed on a 24-in. screen. The numbers are 2 cm high in white font with a black background. The distance is 0.8 m and the viewing angle is 1.001. The evaluation process is shown in Fig. 3.

#### Secondary outcome measures

##### Response time (RT) value of visualizing 1-back digital tasks

The response time usually represents the time for the subject to respond to the stimulus and is closely related to the patient’s attention. The way that select and analysis the RT value (RT-hit) is the most commonly method to evaluate the response.

##### The Rivermead Behavioral Memory Test (RBMT)

RBMT-II is mostly used to evaluate memory recovery in patients with brain injury. The test operation is simple, and the entire test process takes 20~30 min. Specific assessments include the followin g[32, 33]: (1) Remembering a person’s name. The detection method is to first show the subject the associated name and face (photo), and then, require the subject to say the corresponding name when showing the photo to him again (without telling the subject in advance that he needs to complete this test). (2) Remember 10 common items (requires subjects to name and learn) and 5 faces. The subjects are first shown some objects and faces, and then ask the subjects to identify the objects and faces that they saw initially after mixing these target stimuli with the same amount of interference stimuli. (3) Remember the main points of a piece of prose, test it immediately, and then test again after 20 min. (4) Find 5 designated points in the room according to the given route, start the test immediately, and then test again after 20 min (for patients with reduced mobility, use a model or a picture to test). At the same time, patients are required to leave a message (envelope) at each point. (5) Remember to ask for their personal items at the end of the test (after 20~30 min). These items were taken by the examiner from the patients at the beginning of the test. (6) Remember a special question (schedule your next appointment). At the beginning of the test, the tester told the patient a special question according to the instructions and then the subject should remember the question when the alarm sounded after 20 min. (7) Ten questions are used to detect the patient’s orientation to time, place, and person. Another question is to ask the patient the date of the day. Each test item is divided into the initial points, and the standard points are finally calculated. The score ranges from 0 to 24. A higher score means better memory function.

##### Trail Making Test (TMT)

TMT is a commonly used neuropsychological test that evaluates subjects’ visual search, scanning, processing speed, thinking flexibility, and executive functions. The trial tracking test consists of two sub-tests: TMT-A and TMT-B. Each sub-test consists of 25 circles distributed on a piece of paper [34, 35]. In the TMT-A evaluation section, the circles are numbered 1–25 and the circles are randomly distributed. Patients should draw lines to connect the numbers in ascending order. In part B, the circle contains both numbers (1–13) and letters (AL); as described in section A, the patient was asked to draw lines connecting circles in ascending order. The difference from part A is that in part B, the patient is asked to draw lines to connect numbers and letters (i.e., 1-A-2-B-3-C) alternately to complete the task. The test requires patients to connect the circles as soon as possible. The score for each section represents the time required to complete the task. The Chinese version of TMT-B uses numeric symbol encoding.

##### Loewenstein Occupational Therapy Cognitive Assessment scale (LOTCA)

This scale includes orientation, perception, visual motor organization, and thinking operation. The orientation includes time and place; perception includes objects, examination of agnosia, apraxia, etc.; visual motion organization includes two-dimensional graphics and three-dimensional building block copying, puzzles, drawing clocks, etc.; and thinking operations include category testing (that is, classification of item pictures), ordering of stories, and graphic reasoning [36]. Finally, the patient’s attention was scored according to his/her overall performance, and the score was calculated into the total score. The lower the score, the more obvious the dysfunction.

##### Modified Barthel Index (MBI)

MBI assesses the impairment of daily activities of patients with neurological disorders and takes the available external support (specific equipment, etc.) into account [37]. The scale rates motor and cognitive functions on a five-point scale (0–4; the higher the score, the less dependent).

##### EEG recording

When patients perform a 1-back task, their EEG signals are also recorded. The EEG signals were recorded with a 64-channel brain electrode (Bp) device at a sampling rate of 1000 Hz. All electrodes have sintered Ag/AgCl sensors that snap directly into the hat (total height 3.5 mm). The impedance recorded in the EEG remains below 8 kΩ. The ground electrode (GND) is located at Fz, and the reference electrode (reference) is located at Cz. A vertical eye movement (VEOG) electrode is located 2–3 cm from the left eye and serves as a reference electrode for online recording. Each patient is required to adjust the sitting position during the entire experiment to keep the whole body muscles, especially the facial muscles relaxed, and try not to move.

##### New blind index

Patient and evaluator blindness will be assessed separately with the new BI [29]. They will be asked which group they think they belong to and choose one of the following answers: verum-EA group, sham-EA group, or unknown. The new blind index is scaled to intervals of − 1 to 1. Among them, 1 means there is no blind method at all, 0 means completely blind method, and − 1 means the opposite guess, which may be related to unblinding.

### Data collection methods and management

Data will be collected on Case Report Forms (CRF), which will resemble the paper forms approved by the ethics committee. The data from primary outcome and secondary outcome will be transferred from the output files and assessment forms to the CRF. An identification code will be allocated for each patient during the enrolment phase to preserve anonymity. Only CRF will be allowed to record patient identification. Two data managers will type the CRF into the computer and check the paper and the electronic data. Except for the ethics committees during audit, all data will be confidential and made anonymous to anyone outside the study. About EEG data, we will solicit participants to consent that the data will be kept at the hospital for future studies. All the data will be available from the corresponding author for use and/or analysis. During the study, If the participants discontinue or deviate from intervention protocols, we will collect as much data as possible for further analysis. Plans that promote participant retention and complete follow-up are as follows: primarily, we will strengthen the communication between researchers and patients, in order to gain the understanding and cooperation of patients as much as possible; furthermore, we will give the patients corresponding financial compensation according to the number of times the patients participated, so as to encourage the patients to continue participating in the experiment until the end of the experiment; last but not least, patients will receive free relevant examination results including cognitive assessment, etc. The results of the experiment will be used to write an article for clinical research, and the article will be published in a relevant journal for professional medical workers and the public to review. The patients will also be informed with the phone number of the attending doctor while undergoing the experiment. It is convenient for them to get more information about the experiment from the attending physician.

### Sample size calculation

The primary endpoint was the difference of accuracy (d`) scores respectively at baseline and endpoint between the three groups (gamma-EA group, theta-EA group, and sham-EA group) in the 1-back test for patients with post-stroke. Since we did not have enough previous research as the basis for our estimated sample size, we chose the data collected in the previous pre-experiment to calculate the sample size required for this experiment. The null hypothesis is that *μ*_*i*_ = *μ*_*j*_ = *μ*_*t*_, while the alternative hypothesis is *μ*_*i*_ ≠ *μ*_*j*_ ≠ *μ*_*t*_, where *μ* is the mean difference and *i*, *j*, and *t* denote the sham-EA, gamma-EA, theta-EA group, respectively. Our pre-experimental results of 1-back accuracy (d`) scores show that after treatment, the d` values of the gamma-EA group (2.48 ± 0.32) and the theta-EA group (2.02 ± 0.53) are improved compared with the sham-EA group (1.88 ± 0.60). Using the sample size calculation formula for comparing the average of multiple independent samples in the parallel design, under the assumptions of α of 0.05 and (1-β) of 0.8, a sample size of 18 per group has been calculated. Considering a 20% dropout rate, a total of 66 patients will be enrolled in the trial (22 per group). The specific calculation formula is as follows [38]: *n* = λ/Δ

- $$ \Delta =\frac{1}{\sigma^2}\sum \limits_{i=1}^K{\left({\mu}_i-\overline{\mu}\right)}^2\kern0.5em \overline{\mu}=\frac{1}{k}\sum \limits_{j=1}^k{\mu}_j $$

### Statistical analysis

#### Baseline comparison

Examine potential baseline differences between the three groups. Chi-square test was used to measure gender, education, and pathological characteristics (intracerebral hemorrhage or cerebral infarction). One-way ANOVA was used to analyze pre-intervention assessment results and information such as age. Baseline comparisons were used to detect potential differences between the intervention and sham-EA group, ensuring that there were no differences between the three groups.

#### The efficacy analysis will use an intention-to-treat analysis

The results of 1-back evaluation are based on the accuracy and response time as the observation indicators. The evaluation results of RBMT, TMT, LOTCA, and MBI are summarized according to the calculation rules of each scale. The results of each of the above assessments are expressed as mean ± SD. The statistical method uses analysis of variance (assuming a normal distribution) and using Kruskal-Wallis (non-parametric test) to test if it does not meet the normal distribution. The significance level was 95% (*p* values alpha < 0.05 on both sides). If the data is missed, the last observation carried forward method will be applied.

#### Specific calculation schemes for the RT value and accuracy of the 1-back paradigm [39]

The RT value in this study is selected for analysis by the RT value (RT-hit), which is the most common method to evaluate the response. Accuracy is represented by the value of d`. The specific formula is as follows: *d* = *Z*_Hit_ − *Z*_FA_. Among them, *Z*_Hit_ and *Z*_FA_ are converted to *Z* values from the hit rate and false alarm rate according to the POZ conversion table. After calculating the *d* value and RT value of each subject, data were imported into SPASS, and one-way ANOVA was used to compare the differences between the three groups before and after the intervention.

#### EEG data

EEG is widely used in neuroscience research [19]. This study is based on previous EEG studies related to WM [25]. Event-related potentials were selected for analysis to explore changes in electrical activity in the cerebral cortex before and after EA intervention at different frequencies. The target components of ERP are N20, P300, and slow-wave NSW. One-way analysis of variance was used and non-parametric tests were used when variances were uneven. Results need to be Bonferroni corrected.

#### New BI

Referring to other blind studies, if the calculated confidence interval of the new BI includes zero, we will consider the blind method to be successful in this study.

Currently, we have not any additional analyses due to smaller sample sizes in each group.

### Patient safety

Adverse events attributable to the EA may include discomfort or bruising at the sites of needle insertion, nausea, or feeling faint. And in severe cases, the needle pain of EA may lead to transient hypertension, inducing recurrence of stroke or cerebral hemorrhage. The method that prevents these adverse events is as follows: (1) Researchers will maintain adequate communication with the patient before the intervention to ensure that the patient will be in a good state before the intervention, including blood pressure and emotional stability, non-hungry and enough rest; (2) During the intervention period, the researchers will pay close attention to the patient’s condition. If the patient feels any discomfort, the intervention will be stopped immediately and the adverse events will be dealt with accordingly. For example, when dizziness and headache occur, intervention should be stopped and the patient will be asked whether they have other discomforts. At the same time, the patient’s blood pressure and heartbeat should be measured immediately, and the attending physician will be notified to diagnose and treat the patient urgently, and if necessary, a corresponding imaging examination will be inspected to the patient; (3) after the patient’s condition is stable, the researcher will follow up patients to record and analyze the causes of adverse events in accordance with the requirements of ethics and clinical trials and discuss prevention plans to reduce the incidence of such adverse events as possible. Any medical occurrence after inclusion (not necessarily caused by the intervention) will be recorded on the CRF and on a progress protocol. This report will be sent to the ethics committee as a safety report. Serious adverse events will be reported to the ethics committee, coordinating center, steering committee, and sponsor immediately, and all participating will be informed by the sponsor, which is the Shanghai Municipal Development Office of Traditional Chinese Medicine. Researchers will try their best to prevent possible adverse events. And if the damage happened, the research team will provide treatment costs and corresponding financial compensation for the patient.

### Organization

Members of the coordinating center include the director, information manager, database programmer, statistical analyst, project coordinator, and research assistants. The director oversees the scientific aspects of the study design. The information managers are responsible for the collection of data and other information and quality assurance activities. Database programmers are responsible for entering data into the database. Statistical analysts are responsible for data analysis. The project coordinators are responsible for study communications, training, and certification of clinical staff in trial procedures. Research assistants are responsible for assisting others. Members of the steering committee include the study chair, data coordinating center director, statistician, and clinical center directors. The responsibilities of the steering committee include the following: (1) review and approval of trial procedures, (2) resolution of technical or operational issues, (3) review of study progress and approval of major changes to the study protocol, and (4) oversight of publication of study findings. In this study, the responsibilities of the endpoint adjudication committee are included in the ethics committee and the data management team is included in the coordinating center. Therefore, there is no independent endpoint adjudication committee and data management team.

### Quality control

To ensure the treatments and assessment at a high standard, both the acupuncturist and the assessor have to provide a proven record of at least 2 years of clinical experience. All clinicians in this study will participate in a 3-day training course in the standard operating procedures provided by the author of the protocol. In this training course, the protocol will be explained and practiced during exercises and role-plays. The coordinating center, independent of the sponsor, will monitor source documents every 3 months. Whether the data is complete and accurate will be determined by the coordinating center. The regulatory binder which includes the protocol and all revisions of informed consent, case report forms, and the investigator’s agreements will be also up to date by the coordinating center.

## Discussion

WM disorder is a hidden dysfunction in post-stroke [4]. In traditional rehabilitation training, there is no effective rehabilitation program for working memory [40, 41]. Acupuncture has an exact effect on the improvement of cognitive function after stroke [42, 43]. However, there is no clear evidence on whether EA can improve WM and the choice of frequency is unclear. Therefore, we design a double center in a random, sham comparison and blinded of patient and evaluator, parallel clinical trial to observe the effectiveness of theta and gamma EA therapy and sham EA therapy for WM.

### Acupuncture points selection

In this study, we chose two acupoints—“Baihui” (GV20) and “Shenting” (GV24), which are situated on the Du meridian—for our study. In the TCM theoretical system of acupuncture, the Du Meridian is the center of the brain and is closely related to the mind [44]. Baihui (DU 20) and Shenting (DU24) are closely related to brain function and are widely used in the treatment of mental and emotional illnesses [45]. Baihui (DU20) is located at the highest point of the head, where all the yang meridians meet. Baihui (GV20) has the functions of clear the mind, lift the spirits, and promote resuscitation in acupuncture [46]. Shenting (DU24) is located on the anterior median line of the head and is also an important acupoint for the Du Meridian. The efficacy of Shenting (DU24) is similar to Baihui. These two acupoints are often used in combination in clinical practice [12]. In modern times, many studies using animal models of stroke have revealed that acupuncture at the Baihui (DU20) and Shenting (DU24) acupoints can ameliorate the learning and memory deficits of rats with cerebral ischemia/reperfusion (I/R) injury [47, 48]. Effects of the treatment include the following: (1) alleviate the damage of brain edema caused by cerebral ischemia [49], (2) increase the expression of cell division cycle [50], (3) increase cerebral perfusion of the cerebral cortex [51], and (4) increase the plasticity of neuroblast and enhancing the proliferation of cell [52].

### Outcome measurements selection

*N*-back task, as the primary outcome, is one of the traditionally been used paradigms to explore the neural basis of WM and executive function (EC) [53]. Neuropsychological studies show EC (including shifting, updating, and inhibition) is a core component of WM [54], unlike other simple storage tasks, such as forward digital span task [55]; *n*-back task is regarded as a typical task loading on WM updating [56]. In *n*-back task, participants need to decide whether the current stimulus is the same as the stimulus they saw *n* steps back. The larger the value of *n*, the higher the difficulty level of the test [57, 58]. In the pre-experiment, we booked 9 patients with cerebral infarction and randomly assigned them to the theta-EA group, gamma-EA group, and sham-EA group. The intervention parameters and course of treatment are consistent with this experiment. Before intervention, patients were evaluated 0-back, 1-back, and 2-back. It was found that 6 subjects had difficulty performing 2-back, so only 0-back and 1-back were evaluated after the intervention. The results showed that 0-back has the ceiling effect. Thus, we choose 1-back to assess patients’ working memory. In addition to dealing with behavioral evaluations, analysis of the EEG signals is also an important aspect. Studies have shown that the different components of ERP are closely related to the information processing process of working memory [59]. For example, N2 is related to the maintenance of information [60], and P3 is related to the updating of information and allocation of attentional resource [61]. Hence, we recorded the task status of the patient’s EEG signals to explore the impact of the intervention scheme on the electrical activity of the cerebral cortex, including relative to sham.

### Limitation

There are some limitations in this protocol. First, because there are few clinical studies on working memory rehabilitation of patients after stroke, this study is based on the results of the pre-experiment to calculate the sample size. The sample size of this study is small, which is insufficient to conduct a comprehensive study. Second, previous acupuncture studies have often used body and scalp acupoints for post-stroke patients with cognitive impairments [62–64]. However, considering that this study will also observe the effect of EA on the electrical activity of the cerebral cortex, we will only use two scalp acupoints to reduce the interference caused by too many acupoints.

## Trial status

Ongoing recruitment.

Protocol version number V1, April 2020. Recruitment began on April 2020 and will be completed by June 2021.

## Data Availability

The datasets used and/or analyzed after completing the current study will be available from the corresponding author by reasonable requests.

## Abbreviations

WM: Working memory
EA: Electroacupuncture
MMSE: Mini-Mental State Examination
RBMT: The Rivermead Behavioral Memory Test
TMT: Trail Making Test
LOTCA: Loewenstein Occupational Therapy Cognitive Assessment Scale
MBI: Modified Barthel Index
new BI: New blind index
